# Integrated management is effective in the prevention and control of COVID-19 in the blood purification center: joint efforts from multiple departments

**DOI:** 10.1186/s41100-021-00334-y

**Published:** 2021-04-12

**Authors:** Jinmei Yin, Jun Yin, Zhiwu Tian, Peiqiu Li, Xiaoqiu Chen

**Affiliations:** 1 Blood Purification Center, The Fifth Affiliated Hospital of Sun Yat-Sen University, Zhuhai, China; 2 Blood Purification Center, The Third Affiliated Hospital of Sun Yat-Sen University, Guangzhou, China

**Keywords:** COVID-19, Multi-department integrated management, Blood Purification Center, Prevention and control

## Abstract

**Background:**

During the outbreak of new coronavirus pneumonia, many hospitals in China became the designated hospitals for the treatment of new coronavirus pneumonia. The goal was to develop rapid and effective prevention and control methods for blood purification centers.

**Research design and methods:**

The medical department, hospital department, nursing department, and blood purification center jointly set up a multi-department integrated COVID-19 prevention and control management team to manage the blood purification center. The efforts included the establishment of the continuous renal replacement therapy (CRRT) team for COVID-19, the integrated training and assessment of medical personnel, the integrated education of patients and their families, and the integrated management of the workflow of the hemodialysis room.

**Results:**

No infected persons, including medical staff, patients, and their families, have been found in the dialysis center. After multi-departmental integrated training, the theoretical performance of medical staff in our dialysis center has increased from 82.36 ± 8.10 to 95.29 ± 4.95 (*p* < 0.05), and the unqualified rate dropped from 23.21 to 1.78% (*p* < 0.05). In addition, the three operational skills evaluation scores have also been significantly improved, from 86.00 ± 4.02, 88.01 ± 6.20, 92.01 ± 2.46 to 95.90 ± 0.30, 97.21 ± 0.87, 96.00 ± 1.00 (*p* < 0.01), and the passing rate from 80.36 to 100% (*p* < 0.05).

**Conclusion:**

Medical staff’s knowledge of novel coronavirus pneumonia prevention and control can be improved by multi-sectoral integrated management, and CRRT treatment of COVID-19 patients is effective.

## Background

In the world, not only for the novel coronavirus pneumonia in people’s work, study, and life had a tremendous negative impact, but also increase the economic burden of disease and panic in the country. As the influence continues to expand, the new coronavirus pneumonia has received increasing attention. The World Health Organization (WHO) named it “COVID-19” [1], and in China, COVID-19 has been included in class B infectious diseases, and measures to prevent and control class A infectious disease have been adopted.

In response to prevention and control requirements, public gathering activities should be minimized to avoid cross-infection [2]. But for patients undergoing maintenance hemodialysis, as a guarantee for the continuation of life, it is necessary to dialyze 2 or 3 times a week [3]. Therefore, the blood purification center has to become a medical place with a high risk of exposure due to its high mobility and relatively dense population.

The Fifth Affiliated Hospital of Sun Yat-Sen University is the only provincial designated treatment hospital for COVID-19 in Zhuhai, China. Its blood purification center is the Zhuhai Blood Purification Quality Control Center, and there are 463 patients on maintenance hemodialysis. Therefore, the prevention and control of COVID-19 in the blood purification center is the top priority of the epidemic prevention process. After multi-departmental joint coordination, the blood purification center has integrated management of patients and medical staff in response to COVID-19 and has achieved good management outcomes, which are summarized as follows.

## Materials and methods

### Build a multi-departmental integrated prevention and control team

Establish a prevention and control working group that is coordinated by the Medical Affairs Department, Hospital Infection Management Department, Nursing Department, Infection Department, and Blood Purification Center, and build a three-level prevention and control model for the director of the department/the chief nurse—medical team leader/nursing team leader/engineer—first-line medical staff/patients and their families. All departments work together to ensure the rationality of the system and the effective implementation of prevention and control measures.

### Management of medical staff

Medical staff use online (DingTalk, WeChat) and offline methods to learn about COVID-19 related clinical knowledge and protective measures, and strictly complete the learning of operational skills such as putting on and taking off protective clothing and nucleic acid specimen collection [4].

The nursing team leader assigns specific personnel to measure and record the body temperature of the medical staff on duty before and after work every day to confirm whether they have travel history in the epidemic area, history of contact with suspicious persons, and symptoms such as fever and vomiting. If relevant suspicious symptoms are found, they should be taken immediately isolation and carry out relevant measures such as nucleic acid testing and lung CT examination.

### Management of dialysis patients and their families

Regularly arrange nucleic acid tests for patients and their families to check for COVID-19 infection, and the nurse in charge conducts epidemiological history investigation and health education on patients based on the self-made “COVID-19 Epidemic Area Contact Questionnaire” and “COVID-19 Prevention and Control Education Sheet.” Patients wear masks throughout the dialysis process, wash their hands with running water before dialysis treatment, and regularly monitor their body temperature by nurses during dialysis [5].

Strengthen the pre-examination and triage of patients, and report suspected infections such as fever and cough to the Hospital Infection Management Department and arrange for relevant treatment in the infectious disease department. After the patient completes blood routine, nucleic acid test, lung CT, and other examinations to rule out COVID-19 infection, then arrange for treatment in the dialysis room.

### Management of dialysis room

The blood purification center has set up a single respiratory isolation area for patients from the epidemic area and those who may be exposed to COVID-19, and relevant medical staff should protect themselves with protective clothing and goggles [6]. After the dialysis is completed, the logistics personnel clean and disinfect the isolation area and common areas of the dialysis room according to the guidelines, including opening windows for ventilation, ultraviolet disinfection and sterilization, and wiping the ground and the surfaces of objects that may be contacted with a chlorine-containing disinfectant [7].

### Outcome evaluation

The outcome evaluation indicators of this study mainly include (1) the incidence of COVID-19. (2) Theoretical knowledge evaluation: before and after the training, all medical staff will conduct a theoretical assessment of COVID-19-related knowledge through questionnaires. There are 25 multiple-choice questions, each with 4 points and a total of 100 points. The specific content includes pathogenic characteristics (1 item) and epidemiological characteristics (3 items), clinical classification (2 items), diagnostic criteria (2 items), treatment plan (4 items), nursing measures (items), hospital infection prevention, and control (7 items). The assessment score is less than 80 as unqualified, 80–89 as qualified, more than 90 as excellent, and colleagues with a score of less than 80 need to make up the exam. (3) Operational skills evaluation: before and after the training, all medical staff passed the on-site operation assessment. Specific items included putting on and taking off protective clothing, nucleic acid specimen collection, and hand washing. The full score for a single skill is 100 points; a total of 300 points. If a single skill is less than 80 points, it needs to be re-tested. The three operational skill assessment scores are all greater than 80 points as qualified, otherwise it is unqualified.

### Statistical analysis

Use SPSS19.0 statistical software for data processing and statistical analysis. Measurement data uses mean and standard deviation, and count data uses frequency and percentage for descriptive statistics; *t* test and chi-square test are used to compare the medical staff’s new crown theoretical results before and after the intervention.

## Results

### Training and management workload

In this special time, our hospital has conducted integrated theoretical and operational training and assessment for staff in batches and conducted integrated health education for patients. The specific workload statistics are shown in Table 1.

**Table 1:** Training and management workload (*n*)

Work project	Number
Total number of theoretical training sessions of staff	12
PowerPoint training course	7
Online lectures via DingTalk	5
Total number of training sessions on staff’s protective operations	68
On-site operation demonstration	7
Video learning	61
Assessment of staff’s theory and personal protective operations	
Number of participants in theoretical assessment	56
Number of participants in operational assessments	56
Patient education and nucleic acid testing	
Number of health education sessions for patients and their families	926
Number of nucleic acid tests on patients’ respiratory tract specimens that showed a negative result	463

### Incidence of infection

There are 56 medical staff in the blood purification center of our hospital, including 11 medical staff for COVID-19 CRRT, 5 industrial staff, and a total of 463 long-term hemodialysis patients. Nucleic acid tests on respiratory tract samples were all negative, indicating that no infection occurred.

### Evaluation of theoretical knowledge and operational skills

After multi-departmental integrated training, the theoretical performance of medical staff in our dialysis center has increased from 82.36 ± 8.10 to 95.29 ± 4.95 (*p* < 0.05), and the unqualified rate dropped from 23.21 to 1.78% (*p* < 0.05) (see Tables 2 and 3). In addition, the three operational skills evaluation scores have also been significantly improved, from 86.00 ± 4.02, 88.01 ± 6.20, 92.01 ± 2.46 to 95.90 ± 0.30, 97.21 ± 0.87, 96.00 ± 1.00 (*p* < 0.01), and the passing rate from 80.36 to 100% (*p* < 0.05) (see Tables 4 and 5).

**Table 2:** Results of theoretical knowledge evaluation ($\overline{x}\pm s$)

Group	Cases (*n*)	$\overline{x}\pm s$	*t* value	*p* value
Before training	56	82.36 ± 8.10	12.93	< 0.05
After training	56	95.29 ± 4.95

**Table 3:** Distribution of theoretical knowledge scores (*n*, %)

Group	Number of cases	Failed (%)	Passed (%)	Excellent (%)
Before training	56	13 (23.21)	32 (57.15)	11 (19.64)
After training	56	1 (1.78)	5 (8.93)	50 (89.29)
*χ*^2^	54.92			
*p*	< 0.05

**Table 4:** Results of operational skill evaluation ($\overline{x}\pm s$)

Items	Group	Cases (*n*)	$\overline{x}\pm s$	*t* value	*p* value
Putting on and taking off protective clothing	Before training	56	86.00 ± 4.02	− 24.23	< 0.01
	After training	56	95.90 ± 0.30		
Nucleic acid specimen collection	Before training	56	88.01 ± 6.20	− 15.85	< 0.01
	After training	56	97.21 ± 0.87		
Hand washing	Before training	56	92.01 ± 2.46	− 12.08	< 0.01
	After training	56	96.00 ± 1.00

**Table 5:** Distribution of operational skill evaluation scores (*n*, %)

Group	Cases (*n*)	Failed (%)	Passed (%)
Before training	56	11 (19.64%)	45(80.36%)
After training	56	0 (0)	56 (100%)
*χ*^2^	12.20		
*p*	< 0.01		

## Discussion

The multi-departmental integrated management has effectively improved the medical staff’s knowledge about COVID-19 prevention and control and has avoided COVID-19 infection incidents in our medical personnel and patients. According to the case analysis of medical staff in the “Analytical Report of Epidemiological Characteristics of Novel coronavirus Pneumonia” issued by the Chinese Centers for Disease Control, a total of 3019 medical staff from 422 medical institutions providing diagnosis and treatment services for COVID-19 patients were infected with SARS-CoV-2; 1716 infections were confirmed, and 5 patients died of the infection, although non-occupational exposure-caused infection might also have occurred [8]. In the early stage of the epidemic, especially in Hubei, there might have been inadequate education on the epidemic, a shortage of protective gear, and insufficient protection awareness and measures of medical personnel, which were important causes of infection. Upon the outbreak of the COVID-19 epidemic, we immediately launched a multi-department integrated management setup in our hospital and quickly set up a COVID-19 prevention and control team. The blood purification center was one of the key prevention and control departments, and the medical personnel in the center actively participated in the integrated training and assessment in the early stage of the epidemic. Their average score on the test of their knowledge about COVID-19 prevention and control increased from 82.36 ± 8.10 before the training to 95.29 ± 4.95 after the training (*t* = 12.93), and the pass rate also increased from 76.79 to 98.22% (*p* < 0.05). At the same time, the center conducted early screenings and integrated health education for patients and their families using the self-developed “Questionnaire on contact with epidemic areas or patients with COVID-19” and “COVID-19 prevention and education pamphlet” so the medical personnel and patients could fully acquire the relevant knowledge on COVID-19 and thus improve their awareness of prevention measures. The *Novel Coronavirus Pneumonia Diagnosis and Treatment Plan (Provisional 6th Edition)* [9] issued by the NHC has made it clear that (1) the sources of infection seen so far are mainly patients with novel coronavirus infection, while asymptomatic individuals with the infection may also become sources of infection; (2) respiratory droplets and close contact are the main transmission routes, while it is also possible that the virus can transmit through aerosol when individuals are exposed to a high concentration of aerosol in a relatively closed environment for a long time; (3) the whole population is susceptible to the virus. Based on the epidemiological characteristics of COVID-19, on the premise of integrated training of doctors and nurses, we conducted integrated management of hemodialysis rooms, formulated the “Protective procedure for the prevention and control of COVID-19 in blood purification centers,” strengthened the pre-examination and triage of patients, set up isolated quarantine rooms for respiratory diseases while strengthening the management of ordinary dialysis areas, and performed strict cleaning and disinfection on the environment and the surfaces of objects according to the requirements of different areas. The improvement of medical personnel’s COVID-19 prevention awareness and knowledge has enabled the timely and effective implementation of the prevention and control procedures in our blood purification center, thereby protecting us from COVID-19 infection.

The multi-department integrated management has effectively prompted the rapid formation of a COVID-19 CRRT team, which has ensured the timely treatment of critically ill patients with COVID-19 causes acute respiratory distress syndrome (ARDS) and septic shock, which are the two leading causes of severe illness. On January 29, a paper in *The Lancet* analyzed the epidemiological and clinical characteristics of 99 cases of COVID-19, of whom 17 patients developed ARDS and 11 patients died of multiple organ failure in a short time [10]. The “Expert consensus on the clinical application of blood purification in emergencies” recommends that for the CRRT treatment of sepsis, early intervention is necessary and can be started within 12–48 h of the diagnosis of septic shock [11]. According to some studies, for patients with ARDS with shock, “restrictive fluid replacement” is recommended to avoid excessive input of crystalline fluid during the process of maintaining blood pressure, as it may aggravate pulmonary edema and hypoxia, so it is recommended to perform fluid balance management as soon as possible and CRRT when necessary [12, 13]. Since the outbreak of COVID-19, under our multi-department integrated management setup, human resources have been reasonably and timely allocated, which not only has met the treatment needs of CRRT for critical COVID-19 patients but also has maintained the normal working order in the center. Moreover, medical instruments and materials have been reasonably allocated, which has ensured the material basis of the treatment. Ultimately, owing to the integrated training, the medical staff in our center quickly understood and met the protection requirements for COVID-19 and performed CRRT on two critically ill patients in a timely and effective manner.

## Conclusion

The outbreak of COVID-19 poses a major threat to public health. As the designated Zhuhai City Blood Purification Quality Control Center and the only provincially designated treatment hospital of COVID-19 in Zhuhai, our center is characterized by the presence of highly mobile medical staff and hemodialysis patients and thus is susceptible to infection incidents. The multi-department integrated management setup implemented in the blood purification center in our hospital has the advantages of timeliness, high efficiency, and standardization, has enabled the successful maintenance of the defense against the COVID-19 epidemic, and is thus worthy of introduction and promotion.

## Data Availability

Not applicable

## Abbreviations

COVID-19: Novel coronavirus pneumonia
NHC: National Health Commission of the People's Republic of China
CRRT: Continuous renal replacement therapy
WHO: World Health Organization
ACE2: Angiotensin I-converting enzyme 2
SARS-CoV-2: Severe acute respiratory syndrome coronavirus 2
CRP: C-reactive protein
PCT: Procalcitonin
ARDS: Acute respiratory distress syndrome
